# Intervertebral Disc Rehydration after Lumbar Dynamic Stabilization: Magnetic Resonance Image Evaluation with a Mean Followup of Four Years

**DOI:** 10.1155/2013/437570

**Published:** 2013-04-29

**Authors:** Li-Yu Fay, Jau-Ching Wu, Tzu-Yun Tsai, Tsung-Hsi Tu, Ching-Lan Wu, Wen-Cheng Huang, Henrich Cheng

**Affiliations:** ^1^Department of Neurosurgery, Neurological Institute, Taipei Veterans General Hospital, Taipei 11217, Taiwan; ^2^School of Medicine, National Yang-Ming University, Taipei 11221, Taiwan; ^3^Institute of Pharmacology, National Yang-Ming University, Taipei 11221, Taiwan; ^4^Department of Ophthalmology, National Taiwan University Hospital, College of Medicine, National Taiwan University, Taipei 10002, Taiwan; ^5^Department of Ophthalmology, New Taipei City Hospital, New Taipei City 241, Taiwan; ^6^Department of Radiology, Taipei Veterans General Hospital, Taipei 11217, Taiwan

## Abstract

*Objective.* To compare the clinical and radiographic outcomes in patients of different ages who underwent the Dynesys stabilization. *Methods.* This retrospective study included 72 patients (mean age 61.4 years) with one- or two-level lumbar spinal stenosis who underwent laminectomy and the Dynesys (Zimmer Spine, Minneapolis) dynamic stabilization system. Thirty-seven patients were younger than 65-year old while the other 35 were older. Mean followup was 46.7 months. Pre- and postoperative radiographic and clinical evaluations were analyzed. *Results.* The mean calibrated disc signal (CDS) at the index level was significantly improved from 60.2 ± 25.2 preoperatively to 66.9 ± 26.0 postoperatively (*P* > 0.001). Screw loosening occurred in 22.2% of patients and 5.1% of screws. The improvement in CDS at index level was seen to be significant in younger patients but not in older patients. Overall, the mean visual analogue scale (VAS) of back pain, VAS of leg pain, and the Oswestry disability index (ODI) scores improved significantly after operation. There were no significant differences in pre- and postoperative VAS and ODI and screw loosening rates between the younger and older patients. *Conclusions.* There is significant clinical improvement after laminectomy and dynamic stabilization for symptomatic lumbar spinal stenosis. Intervertebral disc rehydration was seen in younger patients.

## 1. Introduction

Instrumented spinal fusion is the treatment of choice for degenerative spondylosis with instability refractory to conservative treatment [1, 2]. Spine surgeons have also used modern biologics such as recombinant human bone morphogenetic protein-2 to increase the rate of spinal fusion in selected patients [3–7]. However, using biologics to enhance spinal fusion has been sometimes reported with complications postoperatively and during followup. Moreover, even autograft has been repeatedly reported with adverse events, such as donor site morbidity. Not to mention that loss of segmental motion and subsequent adjacent segmental degeneration have also been concerned for the spinal fusion surgery [8–10].

In the recent years, there is the emerging option of dynamic stabilization to spare spinal fusion and still yield satisfactory outcomes in the surgical management of lumbar spondylosis and back pain. Fischgrund and colleagues reported application of the Dynesys (Zimmer Spine, Minneapolis, USA), a pedicle-based lumbar dynamic stabilization system, as an effective alternative to treat lumbar spondylosis in 1994 [11–16]. Theoretically Dynesys can unload the intervertebral disc while providing a restricted range of motion and thus alleviates symptoms in the indexed level of spine. Although its long-term effect on the adjacent segment is still uncertain, there are reports demonstrating improved clinical outcomes with acceptably low rate of screw loosening when Dynesys is used in degenerative disc diseases (DDD), lumbar stenosis, and some spondylolisthesis [13, 17]. Despite the common nature of spondylosis in the diseases treated, quite some disparity exists among these patients, who have variable age and bone quality that could affect the screw anchoring in the nonfused constructs. However, there is a paucity of data addressing the differences of age and clinical outcomes in these patients treated by dynamic stabilization with Dynesys.

This study aims to compare the clinical and radiographic outcomes in patients of different ages who underwent surgical decompression and Dynesys stabilization. This is the first study focusing on the discrepancies between the elderly and the younger patients. The pre- and postoperative magnetic resonance imaging (MRI) especially of each patient were compared to evaluate the condition of the discs at the index segments. All clinical data was prospectively collected and outcomes were measured by standardized parameters with more than two years of followup.

## 2. Methods

### 2.1. Patient Enrollment

From September 2007 to August 2009, the study enrolled 88 consecutive patients who underwent surgical decompression and dynamic stabilization with Dynesys (Zimmer Spine, Minneapolis, MN) in the authors' service. The clinical and radiological data were collected prospectively and then analyzed retrospectively. Three experienced spine surgeons performed all operations in a very similar fashion by the same technique.

The inclusion criteria were the patients who had lumbar DDD with persistent symptoms such as intermittent claudication, low back pain, buttock pain, leg pain, or any combination of the above. Each patient underwent preoperative MRI to confirm the diagnosis and had failed medical treatment for longer than 12 weeks. The exclusion criteria were degenerative scoliosis, prior lumbar surgery, disc diseases requiring discectomies, and spondylolisthesis worse than grade II. Patients with psychiatric disorders, cerebral vascular accidents, coexisting cervical or thoracic myelopathy, or neoplasms were also excluded. Among the 88 patients enrolled in this cohort, 72 completed the follow up for more than 24 months and were thus analyzed. These patients were divided into two groups according to the age at operation: young age (<65 years) and old age (≧65 years).

### 2.2. Operative Technique

Patients were placed in prone position under general anesthesia. Fluoroscopy was used routinely before sterilization to confirm the treated level and keep the lumbar spine in a neutral lordotic curve. Prophylactic antibiotics were infused thirty minutes before incision. After midline incision by subperiosteal dissection, the laminae and spinous process were exposed. The facet joints capsules were preserved intact. Standard total laminectomies and foraminotomies with resection of hypertrophic spur and ligamentum flavum were performed carefully to preserve the complex structure of the facet joints. The exiting and traversing nerve roots were probed through to confirm the adequate decompression without any bony fragment. Bilateral fascial incisions were made by subdermal dissection through the same wound. Along the Wiltse and Spencer intermuscular plane, the titanium alloy screws were placed transpedicularly without destruction of the facet joints [18]. The modular spacers were cut appropriately and the tension cords were assembled to reach 300 N for dynamic stabilization. Fluoroscope was used to assure the correct position of each screw. Drainage catheters were left and the wound was closed layer by layer.

### 2.3. Clinical Assessment

The Oswestry disability index (ODI) scale was used for functional assessment. The 0–10 visual analogue scale (VAS) was used for back and leg pain evaluation. The patients themselves completed the ODI and VAS questionnaire preoperatively and at 1.5-, 3-, 6-, 12-, and 24-month followup regularly. Preoperative scores were compared to postoperative scores.

### 2.4. Radiographic Assessment

Standard anterior/posterior and lateral radiographs, computed tomography (CT), and magnetic resonance imaging (MRI) studies were routinely performed in preoperative assessment. Radiographs were taken regularly at 1.5-, 3-, 6-, 12-, and 24-month followup.

**Figure 1:**
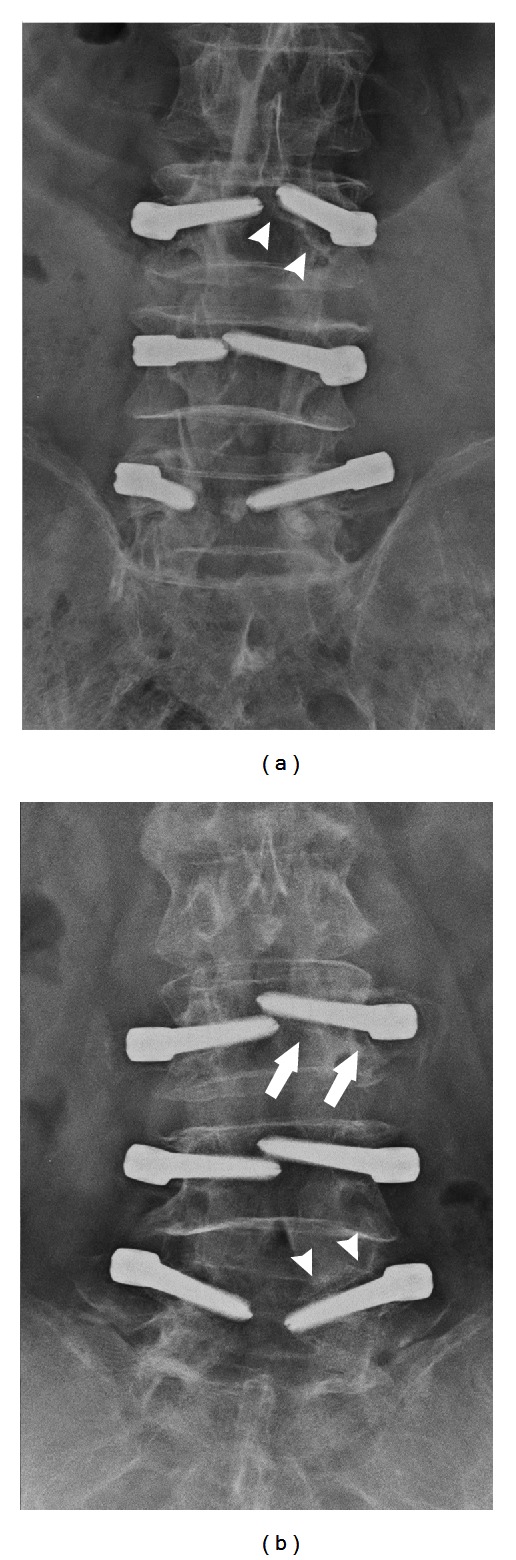
Anteroposterior radiographs. (a) A 61-year-old female who underwent Dynesys stabilization at 12-month postoperation. The double halo sign indicated loosening of the left L3 pedicle screw. (b) A 75-year-old male who underwent Dynesys stabilization, at 2-year postoperation. The double halo sign indicated screw loosening of the left L5 and the halo sign indicated screw loosening of the left L3 screws. Arrowhead, double halo sign; arrow, halo sign.

The presence of “halo zone sign” or “double halo sign” on anteroposterior radiographs was defined as screw loosening during followup (Figure 1). CT was used to determine questionable screw loosening. The halo zone sign was defined as a radiolucent zone alone the screws with 1 mm in width and in any length. The double halo sign was further defined as a radiolucent zone with a radiopaque rim alone the screws.

**Figure 2:**
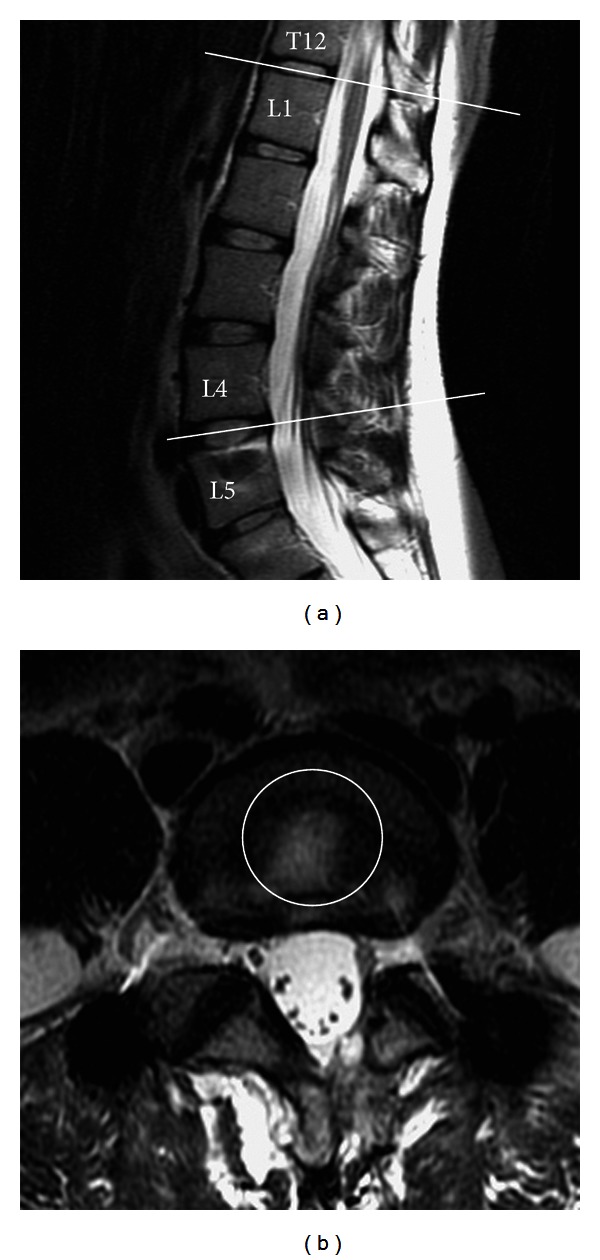
MRI T2-weighted image of a 34-year-old female. (a) Sagittal view. (b) Axial view. The axial view of each disc was retrieved in correspondence with the center of each disc in sagittal view. A circle of one centimeter in radius, region of interest (ROI), was drawn in the geometrical center of each disc. The mean signal intensity of ROI was then measured and recorded. The mean signal intensity of ROI of each level (e.g., L4-5) divided with reference level (i.e., T12-L1) was called CDS.

Pre- and post-operative MRI images were compared focusing on the condition of the discs, including the indexed and adjacent segments. Due to the lack of an absolute unit of measuring the signal intensities on MRI, for example, the Hounsfield unit used in computed tomography to describe radiodensity, these signals of the same patient are difficult to compare. Ideally the pre- and post-operative MRI studies need to be taken by the very same MRI machine to unify the signal intensity references. We tried to allocate the same MRI machine to each patient; however, not every patient achieved the goal. To address this issue, the digital signal in central intervertebral disc of the levels T12-L1 in T2-weighted image (T2WI) was designated as a reference (Figure 2). The signal intensity in the center of the discs in the lumbar spine of each patient was recorded and compared to each patient's own signal intensity recorded at the levels of T12-L1 in T2-weighted MRI (T2WI). The ration differences of signal intensity were calculated and defined as the calibrated disc signal (CDS). The pre- and post-operative CDS of bridged levels were thus all compared and analyzed.

### 2.5. Statistical Analysis

Clinical and radiographic assessments were compared and analyzed using Student's *t*-test, chi-square, and Mann-Whitney *U* test, where appropriate. All data analysis was processed with the statistical software, SPSS version 17.0 (SPSS, Inc., Chicago, IL). Statistical significance was set at *P* value < 0.05.

## 3. Results

Of the 88 consecutive patients with lumbar spondylosis who underwent 1- or 2-level dynamic stabilization with the Dynesys system, 72 patients (81.8%), in whom 370 screws were placed, completed the clinical and radiological evaluations for at least 24 months postoperatively. There were 37 men (51.4%) and 35 women (48.6%) whose mean age was 61.4  ± 11.3 (31–82) years at the time of surgery (Table 1).

The mean followup duration was 46.7 ± 7.8 (33–58) months. In the 72 patients, 31 (43.1%) underwent a 1-level and 41 (56.9%) underwent a 2-level stabilization. The distributions of index levels were as follows: 3 (4.2%) at L3-4; 23 (31.9%) at L4-5; 5 (6.9%) at L5-S1; 1 (1.4%) at L2-3-4; 33 (45.8%) at L3-4-5; and 7 (9.7%) at L4-5-S1 (Table 2).

Overall there were 15 (20.8%) patients diagnosed of type 2 (diabetes mellitus) DM and 9 (12.5%) patients of hypertension. There were more diabetic patients in older age group (34.3%) than in young age group (8.1%) in current study (*P* = 0.006). The hypertensive patients in both groups had similar prevalence rates (10.8% versus 14.3%, *P* = 0.656) (Table 3).

### 3.1. Clinical Outcomes

The overall mean VAS for back pain improved with statistical significance, from 6.3 ± 3.3 preoperatively to 2.8 ± 2.9 postoperatively (*P* < 0.001). There was significant improvement in both the young age group (6.1 ± 3.4 preoperatively to 2.9 ± 2.9 postoperatively; *P* < 0.001) and the old age group (6.4 ± 3.1 preoperatively to 2.7 ± 2.9 postoperatively; *P* < 0.001) (Table 3). There were no significant differences in pre- and postoperative VAS between the young and old age groups (*P* = 0.811 and *P* = 0.754, resp.) (Figure 1).

The overall mean VAS for leg pain improved with statistical significance, from 7.0 ± 2.7 preoperatively to 2.6 ± 3.2 postoperatively (*P* < 0.001). There was significant improvement in both the young age group (6.7 ± 3.0 preoperatively to 2.7 ± 3.1 postoperatively; *P* < 0.001) and the old age group (7.4 ± 2.3 preoperatively to 2.4 ± 3.2 postoperatively; *P* < 0.001) (Table 3). There were no significant differences in pre- and postoperative VAS between the young and old age groups (*P* = 0.497 and *P* = 0.474, resp.) (Figure 1).

The overall ODI functional scores significantly improved from 51.1 ± 19.6 preoperatively to 23.4 ± 21.4 postoperatively (*P* < 0.001). The young age group specifically improved from 50.4  ±  21.3 preoperatively to 21.8 ± 22.0 postoperatively (*P* < 0.001) and the old group from 51.7  ± 17.8 preoperatively to 25.2 ± 20.8 postoperatively (*P* < 0.001) (Table 3). There were no significant differences in pre- and postoperative ODI scores between the young and old groups (*P* = 0.612 and *P* = 0.291, resp.) (Figure 1).

### 3.2. Radiographic Outcome of Screw Loosening

There was screw loosening in 16 out of 72 patients (22.2% per patient) and 19 out of 370 screws (5.1% per screw). Specifically, there were 8 of 37 patients (21.6%) and 9 of 188 screws (4.8%) found loosened in the young age group. In contrast, there were 8 of 35 patients (22.9%) and 10 of 182 screws (5.5%) found loosened in the old age group. There was no statistically significant difference found between the two groups in the rates of screw loosening per patient or per screw (*P* = 0.900, *P* = 0.739, resp.) (Table 4).

### 3.3. Radiographic Outcome of Bridged Disc Signal

**Figure 3:**
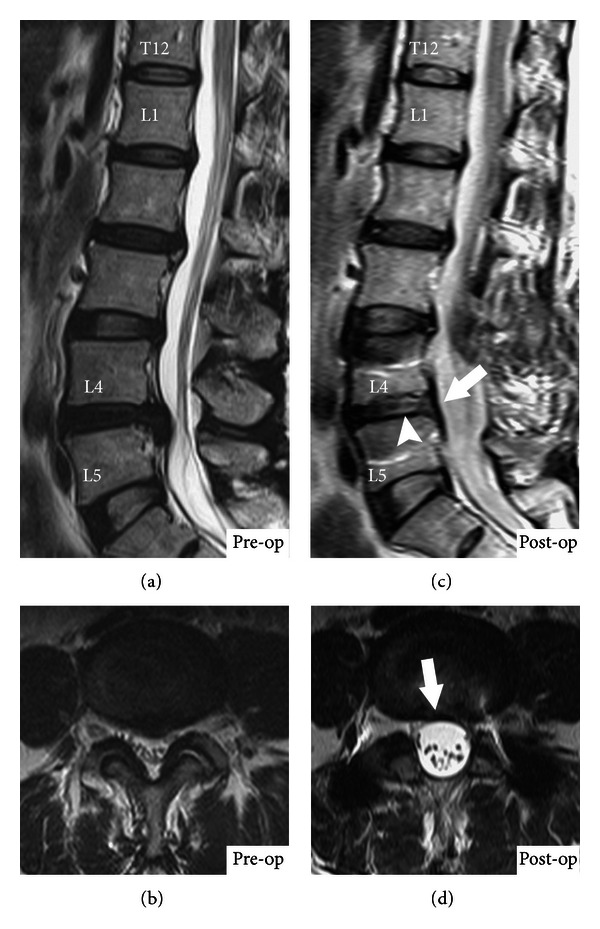
MRI T2-weighted image of a 56-year-old female. ((a) and (c)) Sagittal view. ((b) and (d)) Axial view. The significant increase in CDS was seen in L4-5 level (preoperative 0.44 to postoperative 0.92) at 24-month followup. Meanwhile, the signal intensity of T12-L1 demonstrated to be similar brightness. Arrow, reduced bulging disc after stabilization; arrowhead, significant increase in CDS.

The mean overall CDS at bridged level improved (i.e., increased) with statistical significance, from 60.2 ±25.2 preoperatively to 66.9 ±  26.0 postoperatively (*P* = 0.014). There was significant increase in the young age group (58.9 ± 24.7 preoperatively to 67.6 ±  28.7 postoperatively; *P* = 0.013) (Figure 3). However, the change in the old age group was not significant (62.1 ± 26.1 preoperatively to 65.9 ± 22.2 postoperatively; *P* = 0.366). There were no significant differences in pre- and post-operative bridged level CDS between the young and old age groups (*P* = 0.732 and *P* = 0.800, resp.) (Table 4).

## 4. Discussion

The current study collected total 72 patients with lumbar spondylosis who underwent decompression and dynamic stabilization. A total 370 screws of Dynesys were placed during the operations. In a mean follow-up period of 46.7 months, the results of both the young age (<65 years old, *n* = 37) and the old age (≧65 years old, *n* = 35) groups demonstrated satisfactory improvement in clinical outcomes. The overall screw loosening rate was 22.2% per patient (16 in 72 patients) and 5.1% per screw (19 in 370 screws). The rate of screw loosening was slightly higher in the old age patients. Regarding disc signal, in the overall CDS of bridged discs, the change significantly improved from 60.2 ± 25.2 preoperatively to 66.9 ± 26.0 postoperatively (*P* = 0.014). However, the change in the young age group (58.9 ± 24.7 to 67.6 ± 28.7, *P* = 0.013) appeared to be more obvious than in the old age group (62.1 ± 26.1 to 65.9 ± 22.2, *P* = 0.366).

Using the 10% difference as minimal clinically important difference (MCID) of the ODI defined by Hägg et al., and the 1.2 unit improvement of VAS reported by Copay et al., overall there were 68.1% (49/72) of patients had MCID of VAS back pain, 84.7% (61/72) had MCID of VAS leg pain, and 81.9% (59/72) had MCID of ODI [19, 20]. Therefore, a significant number of patients had clinically significant improvement in the present series at a mean followup of 47 months.

According to the population projections of the United Nations, the number of elderly patients (older than 65 years) in the world will increase from 8 to 14 percent and the percentage will increase far more to 25 percent in more developed nations between 2010 and 2040. Low back pain and lumbar spondylosis is a common problem in the elderly patients which may greatly affect their quality of life. Pain, numbness, claudication, and risk to fall are the frequent symptoms of the patients. The prevalence of lumbar spondylosis will increase as the population ages [21, 22]. Conservative treatment such as medication, physical therapy, or manual therapy may be the first-line treatment for most patients and surgical intervention can still achieve satisfactory improvement in selected patients refractory to conservative treatment [23]. Weinstein et al. conducted a cohort study, spine patient outcome research trial (SPORT), to analyze the pain improvement and functional outcomes. They enrolled a randomized cohort of 304 patients and an observational cohort of 303 patients for analysis. They concluded that patients with degenerative spondylolisthesis and spinal stenosis treated surgically, decompression with or without fusion, would maintain greater pain relief and functional improvement [24].

Regarding the influence of age, Deyo et al. reported that complications associated with procedures were primarily related to patients' age [25]. The mortality and morbidity, length of hospitalization, and hospital charge all increased with the ages of the patients. Wang et al. conducted a retrospective study to analyze the clinical outcomes and complications associated with lumbar stenosis surgery in elderly patients (>75 years) in 2003. Eighty-eight patients older than 75 years of age who underwent lumbar spondylosis surgery were collected and fifty-two (59.1%) patients received spinal fusion. Among them, 76% experienced complete or partial improvement of back pain, 85% experienced complete or partial relief of leg pain, and 61% of 33 patients with preoperative gait disturbance experienced at least one point on the ambulatory scale. They concluded that lumbar spinal surgery could be conducted safely and with satisfactory outcomes as in young age patients [26]. For more complicated spinal degeneration, Daubs et al. surveyed forty-six patients older than 60 years of age in 2007. These elderly patients with mean age of 67 years underwent spinal deformity surgery to perform thoracic or lumbar arthrodesis procedures of 5 levels or more. The overall complication rate is 37%, including dura tear in 4, iliac vein injury in 4, misplaced pedicle screw in 1, and nerve root injury in 1 patient. The overall mean ODI improvement is 24 points which is statistically significant (*P* < 0.001). They concluded that increasing age was a predicting factor for complication. However, the presence of complications had no association with final clinical outcomes [27].

Dynamic stabilization, Dynesys, is an alternative surgical treatment for lumbar spondylosis. It aims to change the mechanical loading of lumbar spinal segments. Several studies have proved the safety and efficacy of dynamic stabilization but there were only few series involving the Dynesys in the elderly patients [12, 13, 15, 16, 28–30]. Di Silvestre et al. reported that to use this pedicle screw-based system in elderly patients with degenerative scoliosis was able to achieve significant improvement of clinical outcome at last followup. In twenty-nine elderly patients, with mean age of 68.5 years, 51.6% of them had improvement in ODI and 58.2% had improvement in Roland Morris score. The mean improvement was 51.7% and 57.8% in VAS for back and leg pain, respectively. The scoliosis and associated spondylolisthesis remained stable at the last followup [31]. Wu et al. conducted a study to investigate risk factor and outcomes associated with screw loosening of Dynesys. They collected 126 patients with more than 24-month followup. Besides diabetes mellitus, they concluded that old age was identified as major risk factors for screw loosening. Screw loosening can be asymptomatic to clinical outcomes [17].

However, there was no comparative study to discuss the outcomes between young and elderly patients. To date, there is a paucity of long-term data for the application of Dynesys in the elderly patients. The current retrospective study specifically aimed to compare the cohort of young to old age patients with lumbar spondylosis who underwent dynamic stabilization. Regarding the low back pain, the prevalence may be quite high and it may be often underestimated due to inconsistent study design and definition. Cho et al. reported the lifetime prevalence of low back pain was as high as 53.8% in the Asian population [22]. In our study, between the young and elderly patients, the preoperative VAS for back pain was the same (6.1 ±  3.4 versus 6.4 ±  3.1, *P* = 0.811). After decompression and Dynesys implant, they both had significant improvement. Regarding the improvement of VAS back pain, 64.9% (24/37) of patients in young age group had MCID and 71.4% (25/35) of patients in old age group had MCID. The treatment of back pain in elderly seems to be as effective as in young patients. The results of VAS leg pain were similar to back pain. Between the two groups, the preoperative VAS for leg pain was the same (6.7 ± 3.0 versus 7.4 ± 2.3, *P* = 0.497). After surgery, 78.4% (29/37) of patients in young age group had MCID and 91.4% (32/35) of patients in old age group had MCID. The elderly group seemed to have much better result than the young age group.

The preoperative ODI scores were similar between the two groups (50.4 ± 21.3 versus 51.7 ± 17.8, *P* = 0.612). Thirty in 37 (81.1%) young patients and 29 in 35 (82.9%) old patients had MCID in ODI. Both groups had significant improvement after surgery.

Ko et al. reported the screw loosening rate was 19.7% per patient and 4.6% per screw at last followup in 2010. They collected 71 patients with a mean age of 59.2 years and concluded that screw loosening was not associated with clinical outcomes. Most patients with screw loosening were more than 55-year old [13]. Later Wu et al. conducted a study of 126 patients with 658 screws in 2011 and they reported the screw loosening rate as 19.8% per patient and 4.7% per screw. The patients with screw loosening may be asymptomatic at last followup as 37 months long. They concluded that hyperglycemia and old age may be the risk factors of screw loosening [17]. In current study the overall screw loosening rate was 22.2% per patient and 5.1% per screw which were similar to previous studies. The screw loosening rate in the old age group was higher than the young age group in per patient (22.9% versus 21.6%) and per screw (5.5% versus 4.8%). Even the difference did not reach statistically significant but the result was consistent with previous reports. The smaller patient numbers and uncertain cut value of age both could affect the *P* value. Further study with larger number of patients will be required for this question.

In concern of the bridged disc degeneration, Kumar et al. collected 32 patients with lumbar spondylosis who underwent Dynesys stabilization and completed 2-year follow-up MRI in 2008. Till now, there were only few in vivo reports to discuss disc change. By using Woodend scores to define the degeneration classification, they found that there was a significant increase in the scores from preoperative 1.95 to postoperative 2.52 (*P* < 0.001) at the treated levels. The data demonstrated progressive disc degeneration at bridged level after dynamic stabilization. The authors concluded that the degeneration may be due to natural disease progression [32]. Regarding this topic, Vaga et al. collected ten patients with low back pain who underwent Dynesys and analyzed the quantification of glycosaminoglycan (GAG) concentration by MRI at followup. The GAG was increased in 61% of bridged level discs and the result suggested that dynamic stabilization was able to stop or partially reverse the degeneration. Interestingly they thought Pfirrmann grading, even achieved excellent intra- and interobserver agreement, is not sensitive enough to detect the disc change in their study [33]. In 2001, Pfirrmann et al. conducted a study for classification of lumbar disc degeneration. They collected 60 patients with 300 intervertebral discs for analysis. They have established a reliable classification on routine T2-weighted MRI [34]. However, the same protocol for MRI evaluation was not practicable in routine clinical followup. Our study evaluated the disc signal on T2WI series in pre- and post-operative imaging study. Regarding the different MRI machines at each study, we used the CDS which referred to compare the target disc signal with the least mobile level. The significant increase in bridged level CDS, which was consistent to Vaga's report, represented the Dynesys to stop and reverse the disc degeneration. In detail, the young patients had much more improvement than the old patients had.

There were limitations to this study. The cohort composition was not strictly uniform. The old age patients had much higher prevalence of type 2 diabetes which may have some influence on the outcomes. Smoking habit, osteoporosis profile, and body mass index may also have some adverse effect on the outcomes. There were also two iatrogenic dura tear in the old age group (5.7%), but no related symptom was recorded. No other complications (i.e. wound infection) or reoperations have happened to date in the current series.

## 5. Conclusions

There was significant clinical improvement after laminectomy and dynamic stabilization with the Dynesys for symptomatic lumbar spinal stenosis in both the young and old age patients. The screw loosening rate was slightly higher in the old age patients. Disc degeneration may stop or reverse in the young age patients but not for the elderly patients. Further studies are needed to evaluate the regenerative effect of dynamic stabilization on the intervertebral discs.

## Figures and Tables

**Table 1:** Clinical and demographic characteristics (*n* = 72).

Characteristic	Value
Gender	
Male	37 (51.4%)
Female	35 (48.6%)
Age (years) mean	61.4 ± 11.3 (31–82)
Follow-up (months) mean	46.7 ± 7.8 (33–58)
Number of instrumented levels	
1-level	31 (43.1%)
2-levels	41 56.9%)

**Table 2:** Distribution of treated levels.

Instrumentation	Level	Number of patients
1-level (*n* = 31)	L3-4	3 (4.2%)
	L4-5	23 (31.9%)
	L5-S1	5 (6.9%)

2-levels (*n* = 41)	L2-3-4	1 (1.4%)
	L3-4-5	33 (45.8%)
	L4-5-S1	7 (9.7%)

**Table 3:** Comparison between young age and old age.

Variables	Total	Age		*P* values
		<65-year old	≧65-year old	
Number of patients	72	37	35	
Age (years)	61.4 ± 11.3	53.0 ± 9.0	70.3 ± 4.8	<0.001*
Gender				
Male	37	23 (62.2%)	14 (40.0%)	0.060
Female	35	14 (37.8%)	21 (60.0%)	
Number of instrumented levels				
One	31	17 (45.9%)	14 (40.0%)	0.611
Two	41	20 (54.1%)	21 (60.0%)	
Mean pre-op scores				
VAS back pain	6.3 ± 3.3	6.1 ± 3.4	6.4 ± 3.1	0.811
VAS leg pain	7.0 ± 2.7	6.7 ± 3.0	7.4 ± 2.3	0.497
ODI (%)	51.1 ± 19.6	50.4 ± 21.3	51.7 ± 17.8	0.612
Mean 24-month post-op scores				
VAS back pain	2.8 ± 2.9	2.9 ± 2.9	2.7 ± 2.9	0.754
VAS leg pain	2.6 ± 3.2	2.7 ± 3.1	2.4 ± 3.2	0.474
ODI (%)	23.4 ± 21.4	21.8 ± 22.0	25.2 ± 20.8	0.291
Serum glucose status				
Type 2 DM	15	3 (8.1%)	12 (34.3%)	0.006*
Euglycemia	57	34 (91.9%)	23 (65.7%)	
Blood pressure				
Hypertensive	9	4 (10.8%)	5 (14.3%)	0.656
Normotensive	63	33 (89.2%)	30 (85.7%)	

Mean values are presented ± SD.

*P < 0.05, statistically significant.

DM: diabetes mellitus.

VAS: visual analogue scale.

ODI: Oswestry disability index.

**Table 4:** Comparison of disc signal change between young age and old age.

Variables	Total	Age		*P* values
		<65-year old	≧65-year old	
Number of patients	72	37	35	
Screw loosening per patient				
Yes	16	8 (21.6%)	8 (22.9%)	0.900
No	56	29 (78.4%)	27 (77.1%)	
Screw loosening per screw				
Yes	19	9 (4.8%)	10 (5.5%)	0.739
No	351	179 (95.2%)	172 (94.5%)	
CDS of bridged disc				
Pre-op	60.2 ± 25.2	58.9 ± 24.7	62.1 ± 26.1	0.732
Post-op	66.9 ± 26.0**	67.6 ± 28.7**	65.9 ± 22.2	0.800

Mean values are presented ± SD.

**P < 0.05, the post-op value compared with the pre-op value demonstrated significant difference.

CDS: calibrated disc signal.
